# LRP6 is a potential biomarker of kidney clear cell carcinoma related to prognosis and immune infiltration

**DOI:** 10.18632/aging.205440

**Published:** 2024-01-15

**Authors:** Liqun Lu, Yan Lei, Yanling Li, Lujuan Wang

**Affiliations:** 1 Hunan Cancer Hospital and the Affiliated Cancer Hospital of Xiangya School of Medicine, Central South University, Changsha, China; 2 Hunan Key Laboratory of Tumor Models and Individualized Medicine, The Second Xiangya Hospital of Central South University, Changsha, Hunan 410011, China

**Keywords:** LRP6, renal cell carcinoma, Wnt/β-catenin signaling pathway

## Abstract

Renal cell carcinoma is the most common and most lethal genitourinary tumor. The causes of renal clear cell carcinoma are complex and the heterogeneity of the tumor tissue is high, so patient outcomes are not very satisfactory. Exploring biomarkers in the progression of renal clear cell carcinoma is crucial to improve the diagnosis and guide the treatment of renal clear cell carcinoma. LRP6 is a co-receptor of the Wnt/β-catenin signaling pathway, which is involved in cell growth, inflammation and cell transformation through activation of the Wnt/β-catenin signaling pathway. Abnormal expression of LRP6 is associated with the malignant phenotype, metastatic potential and poor prognosis of various tumors. In this study, we found that LRP6 was abnormally highly expressed in a variety of tumors and significantly correlated with microsatellite instability, tumor mutation burden, and immune cell infiltration and immune checkpoint expression in a variety of tumors. Moreover, we found that LRP6 was significantly associated with the prognosis of renal clear cell carcinoma. Further we found a significant correlation between LRP6 and the expression of m6A-related genes and ferroptosis-related genes. Finally, we also found a significant correlation between the expression of LRP6 and the sensitivity to common drugs used in kidney clear cell carcinoma treatment. These results suggest that LRP6 is likely to be a potential target for kidney clear cell carcinoma treatment.

## INTRODUCTION

Renal cell carcinoma (RCC) is a common solid tumor of the urinary system, the tumor mostly originates from renal tubular epithelial cells [1]. The incidence of RCC is increasing gradually annually [2]. There are many histologic types of RCC, including clear cell carcinoma, papillary carcinoma, smoky cell carcinoma, etc., [3]. Among them, clear cell renal cell carcinoma (ccRCC) is the most common, accounting for about 70–75% of all cases [4]. A significant proportion of patients with metastatic ccRCC will have a 5-year survival rate of no more than 10% [5, 6]. The development of targeted drugs can be considered a milestone in the treatment of malignant tumors. The application of targeted drugs in ccRCC has brought a ray of hope to some patients with advanced ccRCC [7–10]. Nevertheless, there is still much room for improvement in the field of targeted drug therapy for ccRCC, and therefore, finding new and more effective therapeutic targets is of great clinical significance for the treatment of ccRCC.

LRP6, one of the many members of the low density lipoprotein (LDL) receptor family, plays a key regulatory role upstream of the Wnt/β-catenin signaling pathway. Typically, LRP6 acts as a co-receptor for the Wnt ligands of frizzled proteins, stimulating downstream signaling that regulates the stability of β-catenin in the cytoplasm. β-catenin can translocate to the nucleus, where it interacts with other transcriptional regulators, thereby influencing the expression of genes critical for cell proliferation, differentiation, and tumorigenesis [11–13]. LRP6 has been reported to have a role in promoting epithelial cell tumors such as hepatocellular carcinoma, colorectal carcinoma, and pancreatic ductal carcinoma, which correlates with tumor malignant phenotype, metastatic potential, and poor prognosis, and is accompanied by increased wnt/β-catenin signaling [13–17]. It has been shown that decreasing LRP6 activity or expression mediates cellular protective mechanisms and inhibits the progression of papillary thyroid and bladder cancers [18, 19].

Systemic treatment for advanced kidney clear cell carcinoma includes radiotherapy, chemotherapy, cytokine therapy, targeted therapy and immunotherapy. Chemotherapy has limited therapeutic effect on metastatic renal cell carcinoma and is often combined with immunosuppressive drugs. With the continuous breakthroughs in targeted therapy and immunotherapy, the treatment of advanced kidney cancer has entered the era of combining targeted therapy and immunotherapy, and the median overall survival of patients has been greatly improved [20–23]. Currently, commonly used targeted drugs for the treatment of advanced kidney clear cell carcinoma include anti-vascular targeted drugs (sunitinib, sorafenib, pezopanib, axitinib, cabozantinib, lenvatinib, bevacizumab, etc.) and anti-rapamycin-targeting protein (mTOR)-targeted drugs (everolimus and tesilomox) [24].

Here, we found that LRP6 is abnormally highly expressed and significantly correlated with microsatellite instability (MSI), tumor mutation burden (TMB), and immune cell infiltration and immune checkpoint expression in a variety of tumors, including renal, breast, colorectal, and lung cancers, etc. Moreover, we found that LRP6 was significantly associated with the prognosis of renal clear cell carcinoma. Further we found a significant correlation between LRP6 and the expression of m6A-related genes and ferroptosis-related genes. Finally, we also found a significant correlation between the expression of LRP6 and the sensitivity to common drugs used in kidney clear cell carcinoma treatment. These results suggest that LRP6 is likely to be a potential target for kidney clear cell carcinoma treatment.

## MATERIALS AND METHODS

### Expression analysis

Expression profiling data for all tumor as well as normal samples were obtained using the The Cancer Genome Atlas (TCGA) database, and bioinformatics was used to analyze the differences in LRP6 expression in all tumor samples and normal tissues.

### TMB and MSI analysis

TMB and MSI data for all tumors as well as normal samples were obtained using the TCGA database, and the correlation between LRP6 expression and TMB and MSI in all tumors was analyzed using bioinformatics.

### Immune cell infiltration and immune checkpoint analysis

Immunocorrelation was assessed for all tumors as well as normal samples using the TCGA database, and the correlation between LRP6 expression and immune cell infiltration in all tumors was analyzed using the CIBERSORT algorithm.

### Prognostic analysis

Expression data of all tumors as well as the corresponding clinical information were obtained using the TCGA database, and univariate Cox regression was used to analyze the correlation between LRP6 expression and prognosis among different tumors.

### Differentially expressed gene, KEGG and gene ontology (GO) analysis

RNAseq data for kidney clear cell carcinoma were obtained from the TCGA database. Differential expression of LRP6 was studied using the Limma package of R software. Adjusted *P*-values were analyzed in TCGA to correct for false positive results. “Adjusted *P* < 0.05 with log2 (fold change) > 1 or log2 (fold change) < −1” was defined as a screen for threshold mRNA differential expression.

To further confirm the potential function of potential targets, the LRP6-regulated differentially expressed genes were analyzed by functional enrichment. GO is a widely used tool for annotating genes with functions, especially molecular functions (MF), biological pathways (BP), and cellular components (CC). KEGG enrichment analysis is practical and can be used to analyze gene functions as well as related high-level genomic functional information.

### Drug sensitivity analysis

LRP6 expression data in each kidney cancer sample were obtained from the TCGA database. The chemotherapy response of each sample was predicted based on the largest publicly available pharmacogenomics database. The half maximal inhibitory concentration (IC50) of one of the samples was estimated by ridge regression to analyze the correlation between LRP6 expression and different drug sensitivities.

## RESULTS

### LRP6 is highly expressed in a variety of tumors

**Figure 1:**
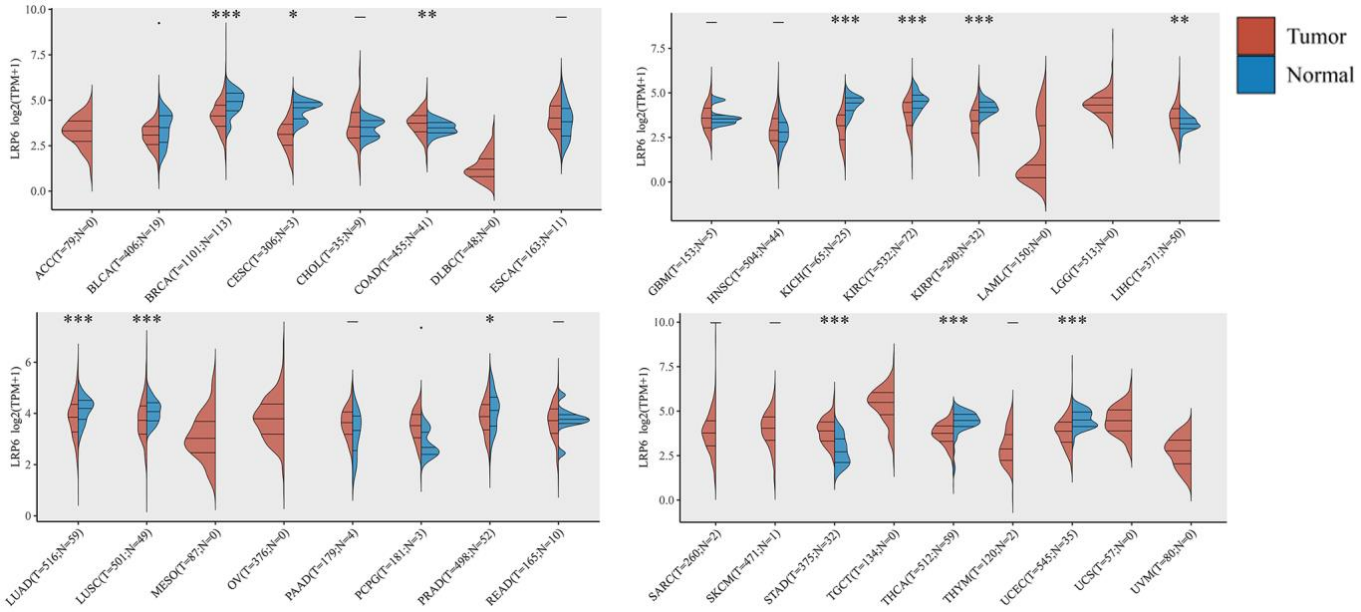
**Expression of LRP6 in pancancer.** The expression distribution of LRP6 in tumor tissues and normal tissues. The abscissa represents different tumor tissues, and the ordinate represents the expression distribution of gene, different colors represent different groups. ^*^p < 0.05, ^**^p < 0.01, ^***^p < 0.001.

LRP6 is an important membrane receptor in the Wnt/β-catenin signaling pathway. To explore the role of LRP6 in tumorigenesis and development, we first analyzed the expression of LRP6 in all tumors using the TCGA database. By comparing the expression of LRP6 in 33 cancer types and their corresponding normal tissues. LRP6 was found to be abnormally expressed in 13 cancer types, including BRCA, CESC, COAD, KICH, KIRC, KIRP, LIHC, LUAD, LUSC, PRAD, STAD, THCA, UCEC (Figure 1). This suggests that LRP6 is likely to play an important role in a variety of tumors.

### LRP6 correlates with TMB and MSI

**Figure 2:**
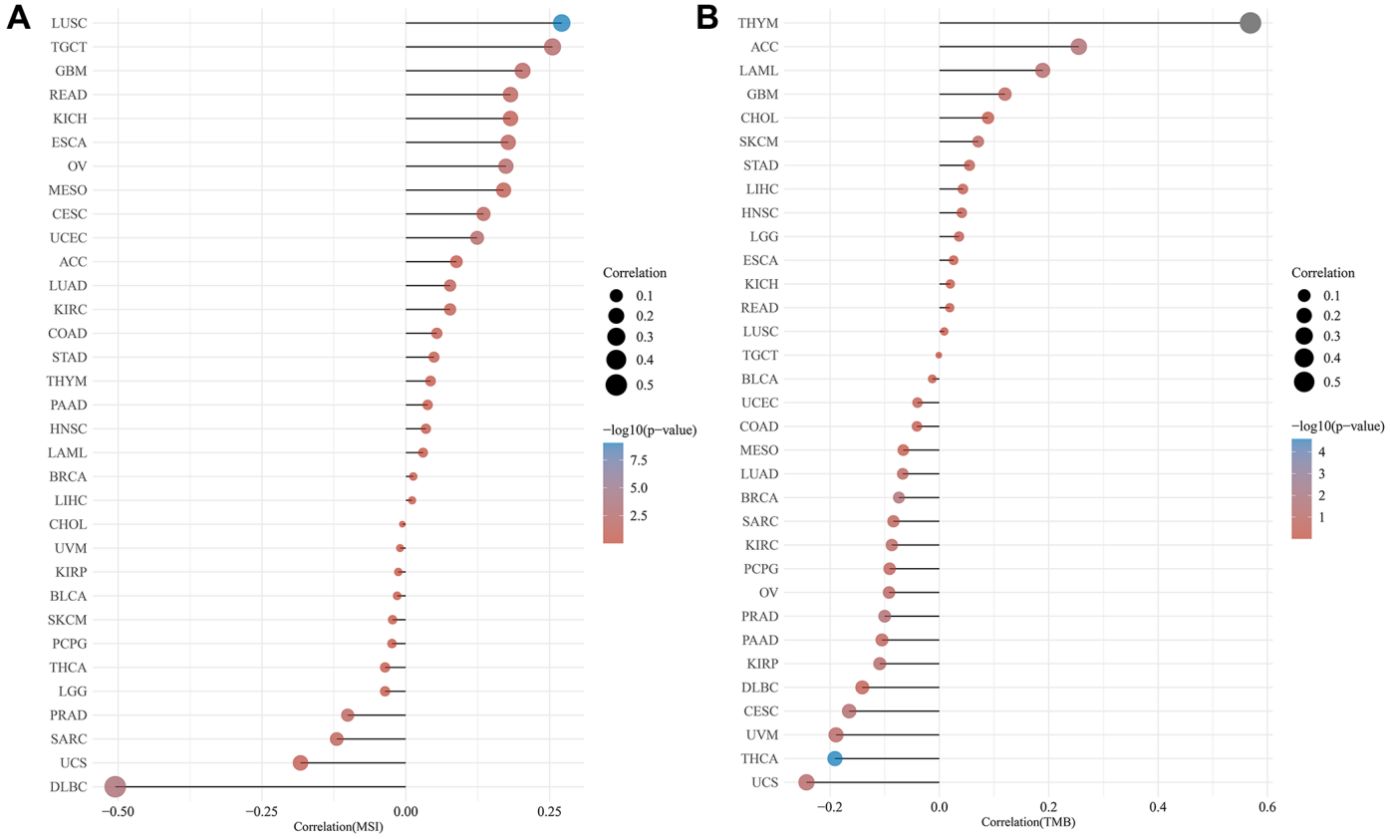
**Correlation analysis of TMB/MSI and LRP6 expression.** The abscissa represents the correlation coefficient between LRP6 and MSI (**A**) and TMB (**B**), the ordinate represents different tumors. The size of the dots represents the size of the correlation coefficient, and different colors represent the significance of *p*-value.

TMB and MSI, not only are important predictors of the efficacy of immunotherapy, but have also been reported to be associated with the efficacy and prognosis of chemotherapy in patients in a variety of tumors [25, 26]. Using data from the TCGA database we investigated the relationship between LRP6 and tumor TMB and MSI, and found that LRP6 expression was significantly positively correlated with tumor TMB in LUSC, TGCT, GBM, READ, and KICH, and significantly negatively correlated with tumor TMB in DLBC, UCS, etc., (Figure 2A). Similarly, we also found that LRP6 expression was significantly positively correlated with the MSI of tumors such as THYM, ACC, LAML, GBM, and significantly negatively correlated with the MSI of tumors such as UCS, THCA, UVM (Figure 2B). These results indicate that LRP6 expression correlates significantly with TMB and MSI, suggesting to us that LRP6 expression may be related to whether tumor patients can benefit from immunotherapy.

### Correlation of LRP6 expression with immune cell infiltration and immune checkpoint expression

**Figure 3:**
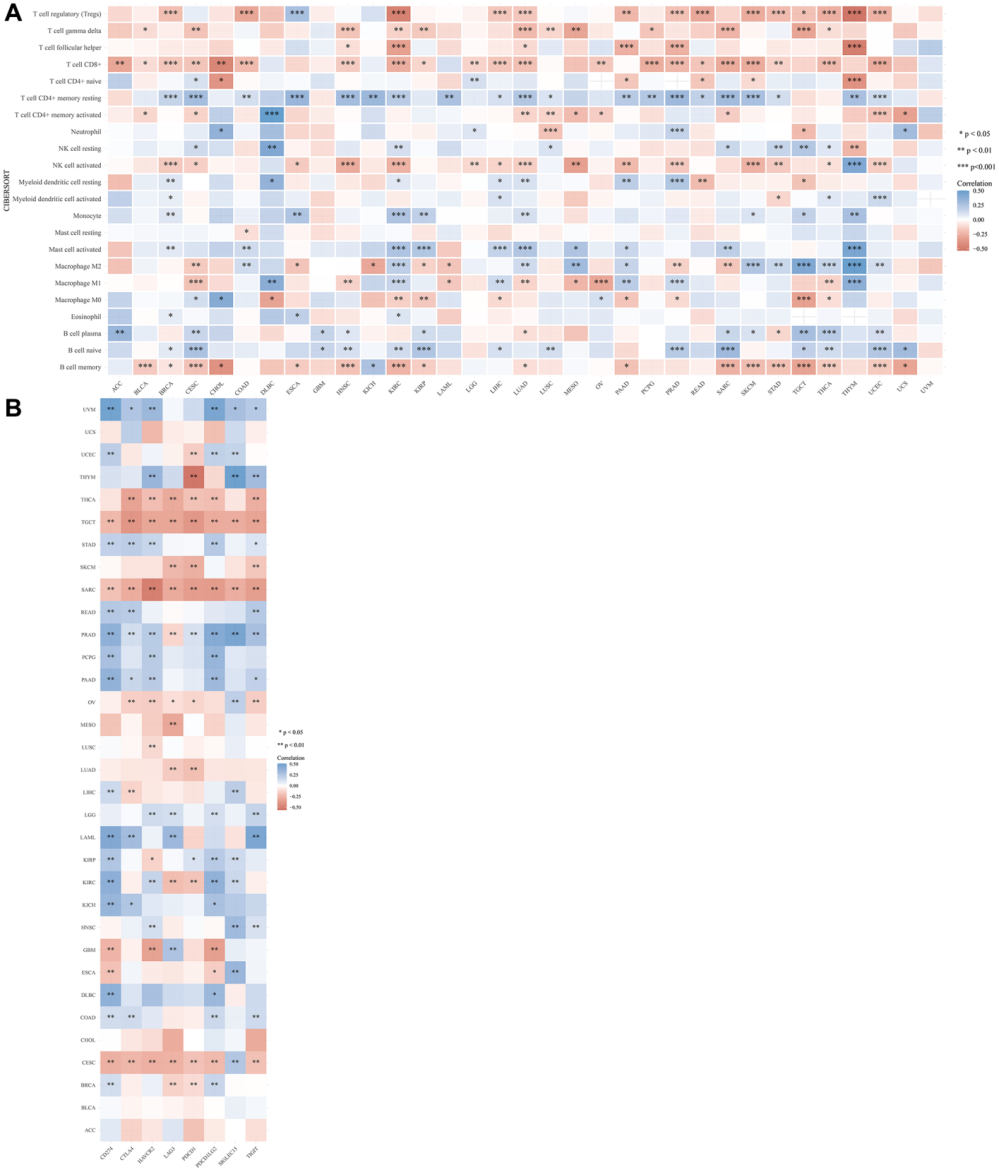
**Correlation between LRP6 expression and various immune cells infiltration and expression distribution of immune checkpoint.** The heatmap of immune score (**A**) and immune-checkpoint-related gene expression (**B**) and LRP6 expression. Each box in the figure represents the correlation analysis between the expression of the LRP6 and the immune score and immune checkpoint in corresponding tumors. ^*^p < 0.05, ^**^p < 0.01, ^***^p < 0.001.

To explore whether LRP6 expression correlates with immune infiltrating cells, we used the CIBERSORT algorithm for analysis. The results showed that the expression level of LRP6 correlated with the infiltration level of immune cells such as T cells CD8+, T cells CD4+, neutrophils, macrophages, dendritic cells, etc., in 33 cancer types (Figure 3A). Subsequently, we also explored the correlation between the expression levels of LRP6 and the expression levels of common immune checkpoints in 33 cancers, such as SIGLEC15, IDO1, CD274, HAVCR2, PDCD1, CTLA4, LAG3 and PDCD1LG2. We found that LRP6 expression was significantly correlated with the expression of immune checkpoints in most of the tumors except UCS, CHOL, BLCA and ACC (Figure 3B). These results suggest that the expression level of LRP6 is closely related to the immune infiltration of various tumors as well as the expression of immune checkpoints.

### LRP6 expression correlates with the prognosis of patients with KIRC

**Figure 4:**
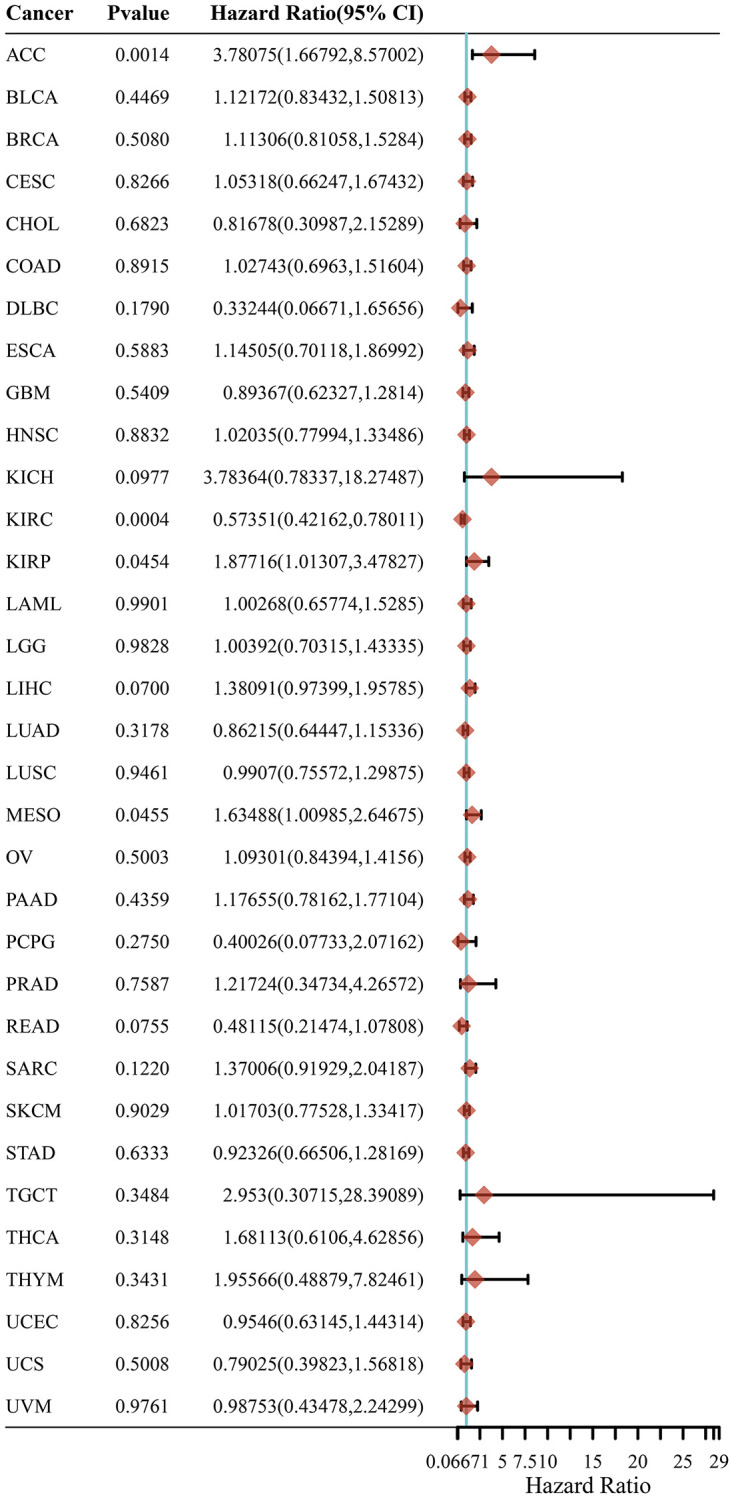
**LRP6 expression correlates with the prognosis of patients with KIRC.** The *p*-value, risk coefficient (HR) and confidence interval of LRP6 in multiple tumours are analyzed by univariate Cox regression.

To test the correlation between LRP6 expression and prognosis of tumor patients, we used univariate Cox regression analysis in order to construct a forest plot and found that LRP6 expression was associated with prognosis in KIRC (HR = 0.574, *p* = 0.0004) (Figure 4). These findings suggest that LRP6 can predict patient survival in certain cancer types and that high LRP6 expression is associated with poor prognosis in kidney clear cell carcinoma.

### Tumor-associated signaling pathways regulated by LRP6

**Figure 5:**
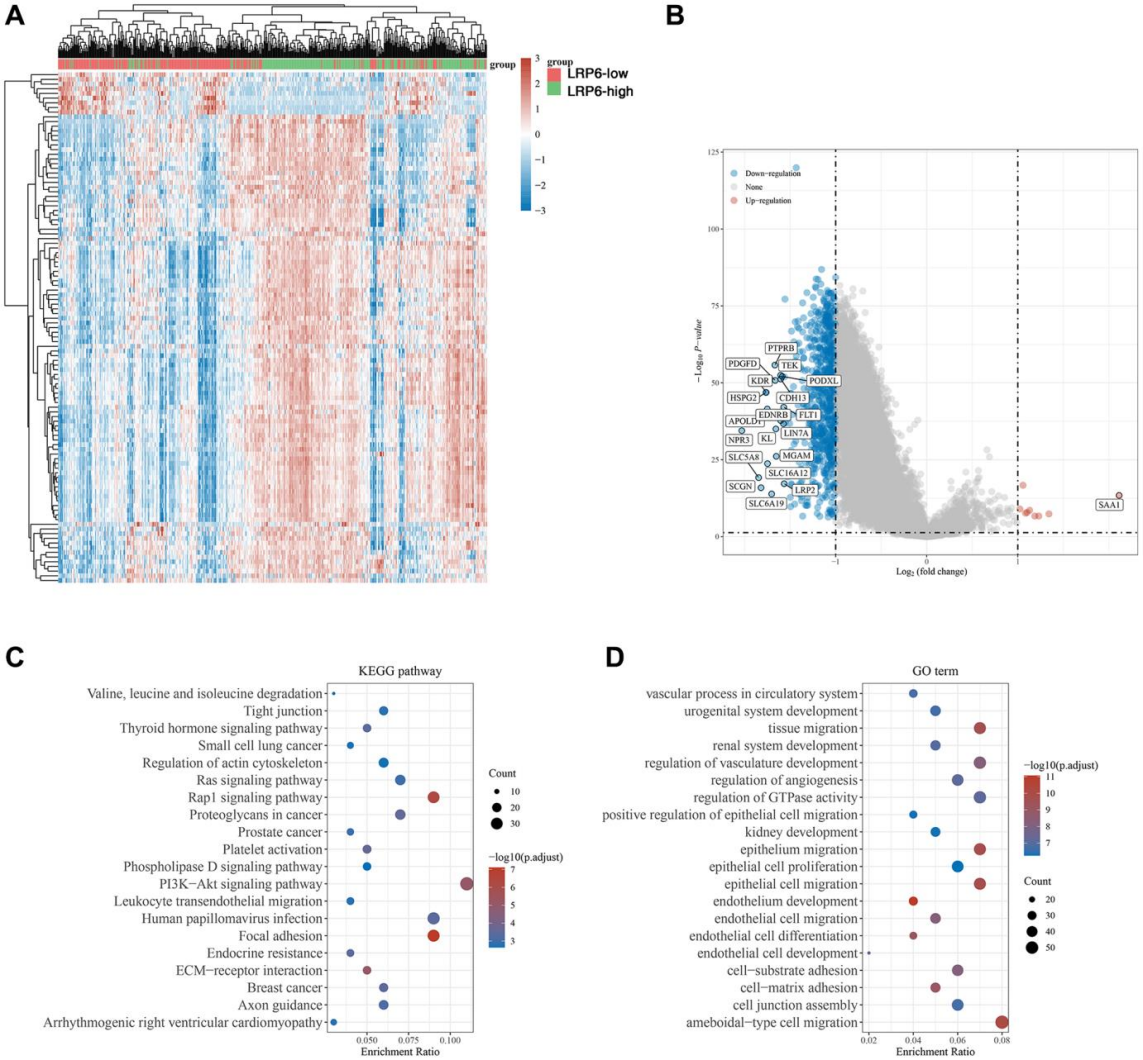
**Biological functions of LRP6 in KIRC samples.** (**A**) Heat map showing differentially expressed genes in KIRC with high and low expression of LRP6. (**B**) Venn diagram showing LRP6-regulated differentially expressed genes. (**C**, **D**) KEGG and GO analyses of LRP6-regulated differentially expressed genes in KIRC.

To explore the mechanisms by which LRP6 may regulate the development of kidney cancer, we first analysed differential gene expression in samples with differential LRP6 expression using TCGA kidney clear cell carcinoma samples, and in total we identified 9 up-regulated and 739 down-regulated genes (Figure 5A, 5B). We further performed KEGG and GO analysis on the differential genes regulated by LRP6 and found that these gene-enriched pathways are closely related to tumor development, such as Ras, ECM, PI3K-AKT and other signaling pathways (Figure 5C, 5D). These results suggest that LRP6 may promote the progression of renal clear cell carcinoma by activating tumor-associated signaling pathways.

### Correlation of LRP6 with m6A-related genes

**Figure 6:**
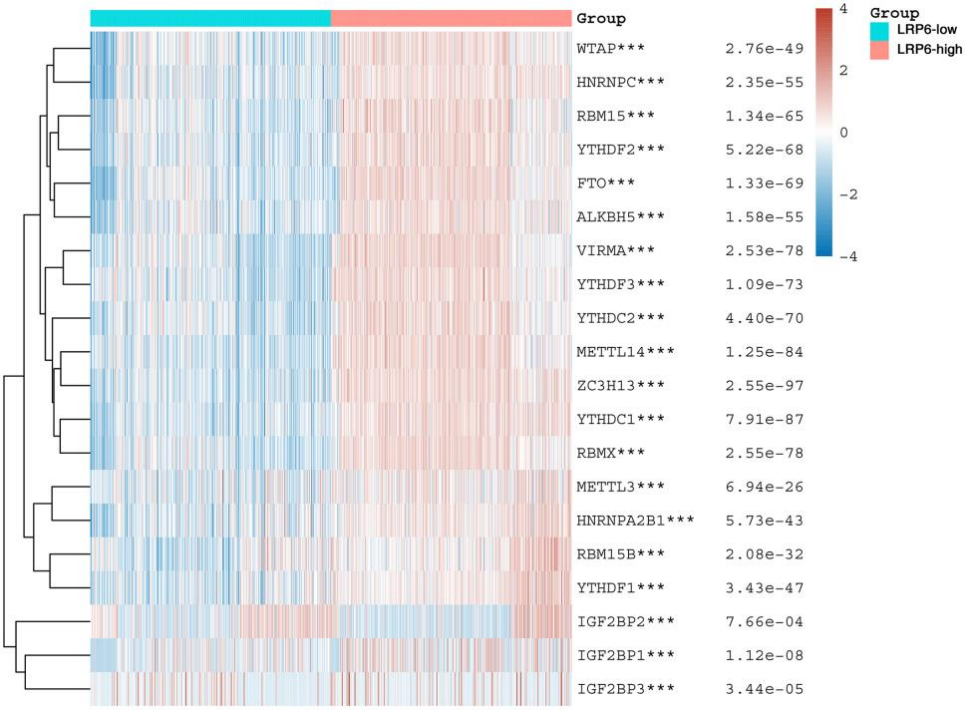
**LRP6 is associated with m6A modification.** Heat map showing the correlation of LRP6 expression with the expression of common m6A-related genes. ^*^stands for significance levels, ^*^p < 0.05, ^**^p < 0.01, ^***^p < 0.001.

Many studies have been conducted on the relationship between m6A methylation and cancer, such as glioblastoma, hepatocellular carcinoma, breast cancer, pancreatic cancer and prostate cancer, etc., and its related mechanisms and functions have been gradually explored [27]. To investigate whether LRP6 may affect renal clear cell carcinoma progression by regulating m6A, we analyzed the correlation between LRP6 and the expression of m6A-related genes, including class “writter” genes (METTL3, METTL14, ZC3H13, VIRMA, WTAP, RBM15 and RBM15B), class “eraser” genes (FTO and ALKBH5), and class “reader” genes (YTHDC1, YTHDC2, YTHDF1, YTHDF2, YTHDF3, HNRNPC, HNRNPA2B1, IGFBP1, IGFBP2, IGFBP3 and RBMX), and found that there was a significant correlation between LRP6 and all 20 m6A-related genes (Figure 6).

### LRP6 is associated with ferroptosis

**Figure 7:**
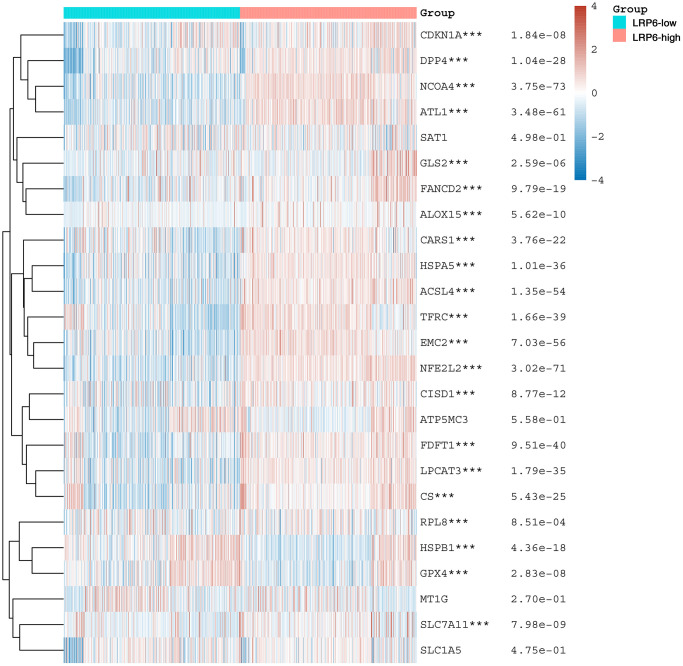
**LRP6 is associated with ferroptosis.** Heat map showing the correlation of LRP6 expression with the expression of common ferroptosis-related genes. ^*^stands for significance levels, ^*^p < 0.05, ^**^p < 0.01, ^***^p < 0.001.

Ferroptosis is a novel mode of programmed cell death caused by excessive accumulation of iron-dependent lipid peroxidation products. In recent years, more and more therapeutic modalities targeting ferroptosis have been developed for anti-tumor therapy [28, 29]. In order to explore whether LRP6 might affect tumor progression through the regulation of ferroptosis, we analyzed the correlation between LRP6 and the expression of 25 common ferroptosis related genes in renal clear cell carcinoma samples, and found that LRP6 was significantly correlated with most ferroptosis related genes except for SAT1, MT1G, and SLC1A5 genes (Figure 7). This suggests that LRP6 may affect the occurrence and development of renal clear cell carcinoma by regulating ferroptosis.

### Expression of LRP6 is correlated with drug sensitivity

**Figure 8:**
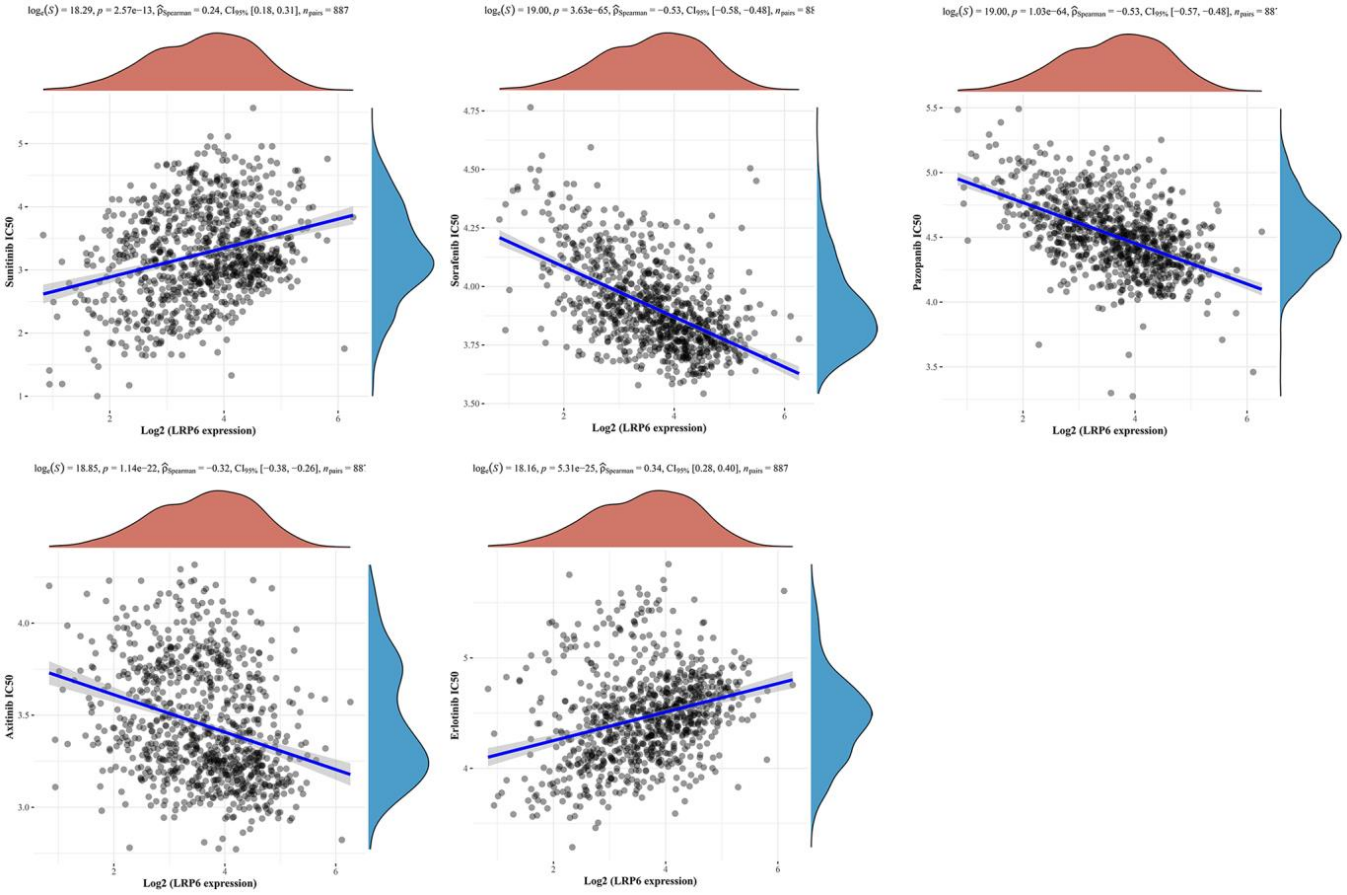
**Correlation between LRP6 expression and drug sensitivity.** Spearman correlation analysis of sunitinib, sorafenib, pazopanib, axtinib and erlotinib IC50 score and LRP6 expression.

To test the association of LRP6 with common drugs currently used to treat KIRC patients, including sunitinib, sorafenib, pazopanib, axtinib and erlotinib, we analyzed the correlation between the expression of LRP6 and drug sensitivity, and found that the expression of LRP6 was significantly positively correlated with the IC50 (half inhibitory concentration) of sunitinib and erlotinib, and significantly negatively correlated with the IC50 of sorafenib, pazopanib, and axtinib (Figure 8). These data implied that LRP6 could be an effective factor in the drug sensitivity of KIRC patients.

## DISCUSSION

Renal cell carcinoma (RCC) is one of the most common urological tumors worldwide, and according to the World Health Organization, there are more than 140,000 RCC-related deaths each year [30]. Among RCCs, clear cell renal cell carcinoma (ccRCC) is the most common subtype, accounting for about 70% to 80% of cases [31]. Patients with early-stage RCC are usually treated with radical nephrectomy or partial nephrectomy and have a favorable prognosis. However, about 25% of RCC patients have metastasis at the time of diagnosis and have a poorer prognosis. In addition, about 20% to 50% of RCC patients will eventually develop metastatic RCC [32]. This progression is closely related to various genes, and it is important to search for potential therapeutic targets. In this study, we found that LRP6 was abnormally highly expressed in a variety of tumors and significantly correlated with microsatellite instability, tumor mutation burden, and immune cell infiltration and immune checkpoint expression in a variety of tumors. Moreover, we found that LRP6 was significantly associated with the prognosis of renal clear cell carcinoma. Further we found a significant correlation between LRP6 and the expression of m6A-related genes and ferroptosis-related genes. Finally, we also found a significant correlation between the expression of LRP6 and the sensitivity to common drugs used in kidney clear cell carcinoma treatment. These results suggest that LRP6 is likely to be a potential target for kidney clear cell carcinoma treatment.

Ferroptosis is a regulated mode of cell death characterized by iron accumulation and lipid peroxidation damage [33]. The process of ferroptosis is characterized by iron overload and accumulation of reactive oxygen species (ROS) generated by lipid peroxidation products. Ferroptosis cells show normal-sized nuclei without chromatin condensation and mitochondrial atrophy and increased membrane density. It can be triggered in cancer cells by depletion of glutathione (GSH) and the amino acid cysteine or by inhibition of glutathione peroxidase 4 (GPX4) [34], but it can be blocked by the iron chelator desferrioxamine (DFO) and by inhibitors of lipid peroxidation, such as ferrostatin-1, liproxstatin-11, etc. Ferroptosis is an intracellular iron-dependent form of cell death, distinct from apoptosis, necrosis, and cellular autophagy [28]. Ferroptosis as a novel and effective cancer therapeutic strategy can influence the efficacy of cancer treatment, and inducing ferroptosis in tumor cells may represent a promising strategy. Here, we found that LRP6 was significantly associated with the expression of ferroptosis-related genes, and whether LRP6 can influence the progression of kidney clear cell carcinoma through the regulation of ferroptosis requires further confirmation.

N6-methyladenosine (m6A) modification is one of the most widely distributed and abundant messenger RNA modifications in eukaryotes. m6A methylation can affect the development of tumor by regulating the expression level of oncogenes or tumor suppressor genes. There are three main types of enzymes involved in m6A methylation modification: methyltransferases, eraser, and reader proteins [35]. Here, we found that LRP6 was associated with the expression of a variety of m6A modification-related molecules, and further confirmation is needed as to whether LRP6 can influence tumor progression by regulating the expression of m6A modification-related genes.

## References

[1] Escudier B, Porta C, Schmidinger M, Rioux-Leclercq N, Bex A, Khoo V, Grünwald V, Gillessen S, Horwich A, and ESMO Guidelines Committee. Renal cell carcinoma: ESMO Clinical Practice Guidelines for diagnosis, treatment and follow-up†. Ann Oncol. 2019; 30:706–20. 10.1093/annonc/mdz056 30788497

[2] Siegel RL, Miller KD, Fuchs HE, Jemal A. Cancer Statistics, 2021. CA Cancer J Clin. 2021; 71:7–33. 10.3322/caac.21654 33433946

[3] Motzer RJ, Jonasch E, Agarwal N, Bhayani S, Bro WP, Chang SS, Choueiri TK, Costello BA, Derweesh IH, Fishman M, Gallagher TH, Gore JL, Hancock SL, et al. Kidney Cancer, Version 2.2017, NCCN Clinical Practice Guidelines in Oncology. J Natl Compr Canc Netw. 2017; 15:804–34. 10.6004/jnccn.2017.0100 28596261

[4] Hanna KS. A review of checkpoint inhibitors in the management of renal cell carcinoma. J Oncol Pharm Pract. 2020; 26:445–58. 10.1177/1078155219881178 31631812

[5] Jonasch E, Walker CL, Rathmell WK. Clear cell renal cell carcinoma ontogeny and mechanisms of lethality. Nat Rev Nephrol. 2021; 17:245–61. 10.1038/s41581-020-00359-2 33144689 PMC8172121

[6] Tan SK, Hougen HY, Merchan JR, Gonzalgo ML, Welford SM. Fatty acid metabolism reprogramming in ccRCC: mechanisms and potential targets. Nat Rev Urol. 2023; 20:48–60. 10.1038/s41585-022-00654-6 36192502 PMC10826284

[7] Au L, Hatipoglu E, Robert de Massy M, Litchfield K, Beattie G, Rowan A, Schnidrig D, Thompson R, Byrne F, Horswell S, Fotiadis N, Hazell S, Nicol D, et al, and PEACE Consortium, and TRACERx Renal Consortium. Determinants of anti-PD-1 response and resistance in clear cell renal cell carcinoma. Cancer Cell. 2021; 39:1497–518.e11. 10.1016/j.ccell.2021.10.001 34715028 PMC8599450

[8] Chen YW, Rini BI, Beckermann KE. Emerging Targets in Clear Cell Renal Cell Carcinoma. Cancers (Basel). 2022; 14:4843. 10.3390/cancers14194843 36230766 PMC9561986

[9] Choueiri TK, Kaelin WG Jr. Targeting the HIF2-VEGF axis in renal cell carcinoma. Nat Med. 2020; 26:1519–30. 10.1038/s41591-020-1093-z 33020645

[10] Makhov P, Joshi S, Ghatalia P, Kutikov A, Uzzo RG, Kolenko VM. Resistance to Systemic Therapies in Clear Cell Renal Cell Carcinoma: Mechanisms and Management Strategies. Mol Cancer Ther. 2018; 17:1355–64. 10.1158/1535-7163.MCT-17-1299 29967214 PMC6034114

[11] Acebron SP, Niehrs C. β-Catenin-Independent Roles of Wnt/LRP6 Signaling. Trends Cell Biol. 2016; 26:956–67. 10.1016/j.tcb.2016.07.009 27568239

[12] Joiner DM, Ke J, Zhong Z, Xu HE, Williams BO. LRP5 and LRP6 in development and disease. Trends Endocrinol Metab. 2013; 24:31–9. 10.1016/j.tem.2012.10.003 23245947 PMC3592934

[13] Raisch J, Côté-Biron A, Rivard N. A Role for the WNT Co-Receptor LRP6 in Pathogenesis and Therapy of Epithelial Cancers. Cancers (Basel). 2019; 11:1162. 10.3390/cancers11081162 31412666 PMC6721565

[14] Garg B, Giri B, Majumder K, Dudeja V, Banerjee S, Saluja A. Modulation of post-translational modifications in β-catenin and LRP6 inhibits Wnt signaling pathway in pancreatic cancer. Cancer Lett. 2017; 388:64–72. 10.1016/j.canlet.2016.11.026 27919787 PMC8005332

[15] Jia Q, Bu Y, Wang Z, Chen B, Zhang Q, Yu S, Liu Q. Maintenance of stemness is associated with the interation of LRP6 and heparin-binding protein CCN2 autocrined by hepatocellular carcinoma. J Exp Clin Cancer Res. 2017; 36:117. 10.1186/s13046-017-0576-3 28870205 PMC5584530

[16] Mao X, Tey SK, Ko FCF, Kwong EML, Gao Y, Ng IO, Cheung ST, Guan XY, Yam JWP. C-terminal truncated HBx protein activates caveolin-1/LRP6/β-catenin/FRMD5 axis in promoting hepatocarcinogenesis. Cancer Lett. 2019; 444:60–9. 10.1016/j.canlet.2018.12.003 30583072

[17] Tung EK, Wong BY, Yau TO, Ng IO. Upregulation of the Wnt co-receptor LRP6 promotes hepatocarcinogenesis and enhances cell invasion. PLoS One. 2012; 7:e36565. 10.1371/journal.pone.0036565 22570728 PMC3343020

[18] Kong W, Yang L, Li PP, Kong QQ, Wang HY, Han GX, Wang QB. MiR-381-3p inhibits proliferation, migration and invasion by targeting LRP6 in papillary thyroid carcinoma. Eur Rev Med Pharmacol Sci. 2018; 22:3804–11. 10.26355/eurrev_201806_15264 29949156

[19] Pierzynski JA, Hildebrandt MA, Kamat AM, Lin J, Ye Y, Dinney CP, Wu X. Genetic Variants in the Wnt/β-Catenin Signaling Pathway as Indicators of Bladder Cancer Risk. J Urol. 2015; 194:1771–6. 10.1016/j.juro.2015.07.032 26173102 PMC5087323

[20] Chang K, Chen Y, Zhang X, Zhang W, Xu N, Zeng B, Wang Y, Feng T, Dai B, Xu F, Ye D, Wang C. DPP9 Stabilizes NRF2 to Suppress Ferroptosis and Induce Sorafenib Resistance in Clear Cell Renal Cell Carcinoma. Cancer Res. 2023; 83:3940–55. 10.1158/0008-5472.CAN-22-4001 37713596

[21] Shah NJ, Sura SD, Shinde R, Shi J, Singhal P, Perini RF, Motzer RJ. Real-world clinical outcomes of patients with metastatic renal cell carcinoma receiving pembrolizumab + axitinib vs. ipilimumab + nivolumab. Urol Oncol. 2023; 41:459.e1–e8. 10.1016/j.urolonc.2023.08.009 37722984

[22] Wu Z, Chen H, Chen Q, Ge S, Yu N, Campi R, Gómez Rivas J, Autorino R, Rouprêt M, Psutka SP, Mehrazin R, Porpiglia F, Bensalah K, et al, and REMEMBER Consortium and the European Association of Urology Young Academic Urologists Renal Cancer Working Group. Prognostic Significance of Grade Discrepancy Between Primary Tumor and Venous Thrombus in Nonmetastatic Clear-cell Renal Cell Carcinoma: Analysis of the REMEMBER Registry and Implications for Adjuvant Therapy. Eur Urol Oncol. 2023. [Epub ahead of print]. 10.1016/j.euo.2023.06.006 37468393

[23] Zhang H, Bai L, Wu XQ, Tian X, Feng J, Wu X, Shi GH, Pei X, Lyu J, Yang G, Liu Y, Xu W, Anwaier A, et al. Proteogenomics of clear cell renal cell carcinoma response to tyrosine kinase inhibitor. Nat Commun. 2023; 14:4274. 10.1038/s41467-023-39981-6 37460463 PMC10352361

[24] Barata PC, Rini BI. Treatment of renal cell carcinoma: Current status and future directions. CA Cancer J Clin. 2017; 67:507–24. 10.3322/caac.21411 28961310

[25] Rizzo A, Ricci AD, Brandi G. PD-L1, TMB, MSI, and Other Predictors of Response to Immune Checkpoint Inhibitors in Biliary Tract Cancer. Cancers (Basel). 2021; 13:558. 10.3390/cancers13030558 33535621 PMC7867133

[26] Schrock AB, Ouyang C, Sandhu J, Sokol E, Jin D, Ross JS, Miller VA, Lim D, Amanam I, Chao J, Catenacci D, Cho M, Braiteh F, et al. Tumor mutational burden is predictive of response to immune checkpoint inhibitors in MSI-high metastatic colorectal cancer. Ann Oncol. 2019; 30:1096–103. 10.1093/annonc/mdz134 31038663

[27] Jiang X, Liu B, Nie Z, Duan L, Xiong Q, Jin Z, Yang C, Chen Y. The role of m6A modification in the biological functions and diseases. Signal Transduct Target Ther. 2021; 6:74. 10.1038/s41392-020-00450-x 33611339 PMC7897327

[28] Jiang X, Stockwell BR, Conrad M. Ferroptosis: mechanisms, biology and role in disease. Nat Rev Mol Cell Biol. 2021; 22:266–82. 10.1038/s41580-020-00324-8 33495651 PMC8142022

[29] Zhang C, Liu X, Jin S, Chen Y, Guo R. Ferroptosis in cancer therapy: a novel approach to reversing drug resistance. Mol Cancer. 2022; 21:47. 10.1186/s12943-022-01530-y 35151318 PMC8840702

[30] Sung H, Ferlay J, Siegel RL, Laversanne M, Soerjomataram I, Jemal A, Bray F. Global Cancer Statistics 2020: GLOBOCAN Estimates of Incidence and Mortality Worldwide for 36 Cancers in 185 Countries. CA Cancer J Clin. 2021; 71:209–49. 10.3322/caac.21660 33538338

[31] Gulati S, Vaishampayan U. Current State of Systemic Therapies for Advanced Renal Cell Carcinoma. Curr Oncol Rep. 2020; 22:26. 10.1007/s11912-020-0892-1 32048058

[32] Mori K, Mostafaei H, Miura N, Karakiewicz PI, Luzzago S, Schmidinger M, Bruchbacher A, Pradere B, Egawa S, Shariat SF. Systemic therapy for metastatic renal cell carcinoma in the first-line setting: a systematic review and network meta-analysis. Cancer Immunol Immunother. 2021; 70:265–73. 10.1007/s00262-020-02684-8 32757054 PMC7889529

[33] Kang R, Zhu S, Zeh HJ, Klionsky DJ, Tang D. BECN1 is a new driver of ferroptosis. Autophagy. 2018; 14:2173–5. 10.1080/15548627.2018.1513758 30145930 PMC6984768

[34] Ursini F, Maiorino M. Lipid peroxidation and ferroptosis: The role of GSH and GPx4. Free Radic Biol Med. 2020; 152:175–85. 10.1016/j.freeradbiomed.2020.02.027 32165281

[35] Sun T, Wu R, Ming L. The role of m6A RNA methylation in cancer. Biomed Pharmacother. 2019; 112:108613. 10.1016/j.biopha.2019.108613 30784918

